# CRISPR Technology for Ocular Angiogenesis

**DOI:** 10.3389/fgeed.2020.594984

**Published:** 2020-12-22

**Authors:** Sook Hyun Chung, Tzu-Ni Sin, Taylor Ngo, Glenn Yiu

**Affiliations:** Department of Ophthalmology and Vision Science, University of California, Davis, Sacramento, CA, United States

**Keywords:** CRISPR, genome editing, retina, angiogenesis, choroidal neovascularization, retinal neovascularization, VEGF, anti-VEGF

## Abstract

Among genome engineering tools, Clustered Regularly Interspaced Short Palindromic Repeats (CRISPR)-based approaches have been widely adopted for translational studies due to their robustness, precision, and ease of use. When delivered to diseased tissues with a viral vector such as adeno-associated virus, direct genome editing can be efficiently achieved *in vivo* to treat different ophthalmic conditions. While CRISPR has been actively explored as a strategy for treating inherited retinal diseases, with the first human trial recently initiated, its applications for complex, multifactorial conditions such as ocular angiogenesis has been relatively limited. Currently, neovascular retinal diseases such as retinopathy of prematurity, proliferative diabetic retinopathy, and neovascular age-related macular degeneration, which together constitute the majority of blindness in developed countries, are managed with frequent and costly injections of anti-vascular endothelial growth factor (anti-VEGF) agents that are short-lived and burdensome for patients. By contrast, CRISPR technology has the potential to suppress angiogenesis *permanently*, with the added benefit of targeting intracellular signals or regulatory elements, cell-specific delivery, and multiplexing to disrupt different pro-angiogenic factors simultaneously. However, the prospect of permanently suppressing physiologic pathways, the unpredictability of gene editing efficacy, and concerns for off-target effects have limited enthusiasm for these approaches. Here, we review the evolution of gene therapy and advances in adapting CRISPR platforms to suppress retinal angiogenesis. We discuss different Cas9 orthologs, delivery strategies, and different genomic targets including VEGF, VEGF receptor, and HIF-1α, as well as the advantages and disadvantages of genome editing vs. conventional gene therapies for multifactorial disease processes as compared to inherited monogenic retinal disorders. Lastly, we describe barriers that must be overcome to enable effective adoption of CRISPR-based strategies for the management of ocular angiogenesis.

## Introduction

Ocular angiogenesis, which is characterized by the formation of new blood vessels from pre-existing vasculature in the eye, underlies the leading causes of blindness across different age groups, including retinopathy of prematurity (ROP), proliferative diabetic retinopathy (PDR), and neovascular age-related macular degeneration (nAMD) (Dreyfuss et al., 2015). Current management of these conditions involve intraocular pharmacotherapies that target vascular endothelial growth factor (VEGF), but are constrained by the variable efficacy and limited durability of these agents. Gene therapies may provide longer term suppression of ocular angiogenesis by hijacking cells in the eye to serve as “biofactories” to produce VEGF antagonists, but their effectiveness remain unclear. Here we discuss the potential for genome editing using Clustered Regularly Interspaced Short Palindromic Repeats (CRISPR) technology for management of ocular angiogenic conditions.

## Ocular Angiogenic Diseases and Therapies

### Retinal and Choroidal Neovascularization

**Figure 1:**
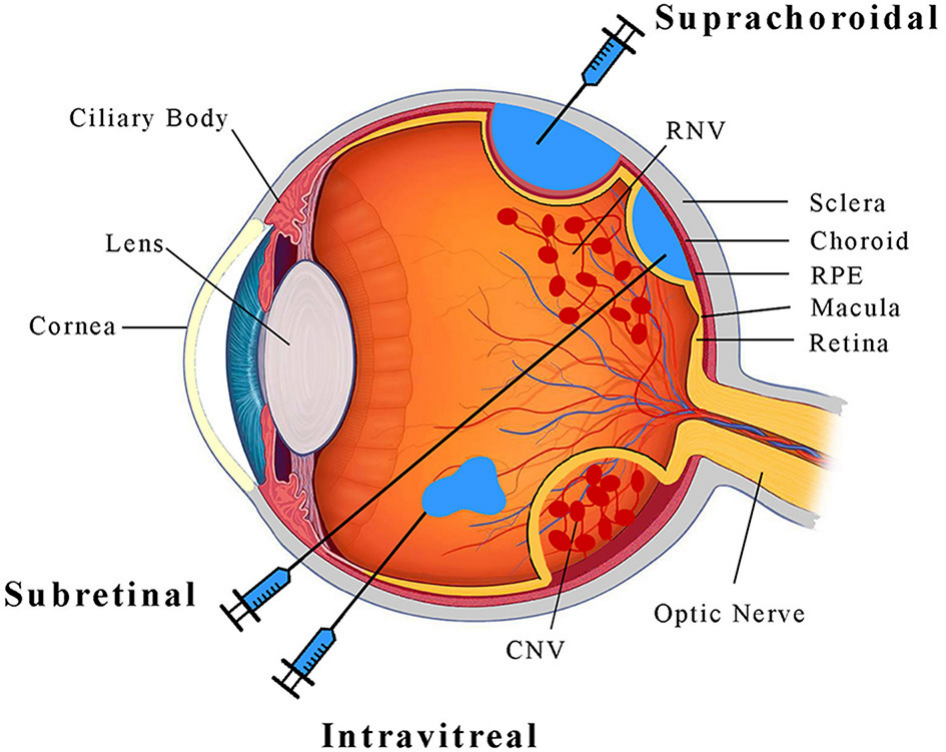
A schematic diagram illustrrating the anatomy of the eye and ocular injection methods.

Pathologic ocular angiogenesis occurs in various parts of the eye, but the two vascular supplies most commonly affected are the retinal and choroidal vasculatures (Figure 1). Retinal vessels arise from the central retinal artery, and supply the innermost layers of the neurosensory retina, such as retinal ganglion cells. Due to their small caliber, retinal vessels are highly susceptible to microvascular diseases such as diabetes mellitus. Diabetic retinopathy is the leading cause of blindness in working-age adults in the United States (Bermea et al., 2018), particularly due to the development of neovascularization in PDR and/or exudation in diabetic macular edema (DME) (Ellis et al., 2019). Another important cause of pathologic retinal neovascularization (RNV) is ROP (Hellström et al., 2013), where early postnatal exposure of premature infants to oxygen delays maturation of the retinal vasculature and leads to retinal ischemia. In both PDR and ROP, the development of RNV leads to retinal and vitreous hemorrhage, fibrovascular proliferation and scarring, and eventually, retinal detachment.

Unlike retinal vessels, the choroidal vasculature, also known simply as the choroid (Figure 1), supplies the outer retinal layers closer to the eye wall. The choroid consists of a spongy meshwork of different caliber vessels that is separated from the retina by the retinal pigment epithelium (RPE) and Bruch's membrane, which together regulate the exchange of nutrients and waste between outer retinal photoreceptors and choroidal vessels, and also serve as part of the blood-retinal barrier. The choroid has the highest blood flow of any organ in the body (Nickla and Wallman, 2010), and creates an oxygen-rich environment which combined with the redox-sensitive lipids of photoreceptors leads to the accumulation of reactive oxygen species with age. In patients with nAMD, this chronic oxidative damage and accumulation of lipid-rich deposits called drusen (Yiu et al., 2020b) can trigger breakdown of the RPE-Bruch's membrane complex leading to choroidal neovascularization (CNV), which can cause subretinal hemorrhage and fibrosis that lead to photoreceptor demise. In pathologic myopia, axial elongation of the globe can similarly lead to breakdown of the RPE-Bruch's membrane barrier to result in CNV and vision loss.

### Molecular Pathways of Ocular Angiogenesis

Pathologic ocular angiogenesis is regulated by multiple angiogenic factors including the VEGF family, platelet-derived growth factors (PDGFs), fibroblast growth factors (FGFs), insulin-like growth factors (IGFs), transforming growth factor- β (TGFβ) superfamily, endothelins, galectins, and integrins (Cabral et al., 2017). Among these, VEGF is considered as the most potent pro-angiogenic factor, as multiple pivotal trials have shown the effectiveness of anti-VEGF therapies in nAMD (Rosenfeld et al., 2006; Brown et al., 2009), DME (Brown et al., 2013), retinal vein occlusions (Brown et al., 2010; Campochiaro et al., 2010; Yiu et al., 2020c), and ROP (Stahl et al., 2019). VEGF is primarily an endothelial cell mitogen. Among its 5 family members (VEGFa-e), VEGFa is the dominant form and considered to be most pathologic, but inhibition of VEGFa alone may trigger compensatory mechanisms from other VEGF isoforms and/or proangiogenic factors (Singh et al., 2015; Cabral et al., 2018). VEGF is secreted by multiple cell types, including endothelial cells, pericytes, RPE, Muller glia, macrophages, and astrocytes (Stone et al., 1996; Miller, 1997; Robbins et al., 1997; Ida et al., 2003). VEGF from Muller cells has been implicated as the major pathologic source in RNV (Wang et al., 2010; Jiang et al., 2014), whereas VEGF from RPEs plays a more significant role in CNV pathogenesis (Kurihara et al., 2012). Thus, although intravitreal agents that globally suppress VEGF are effective in treating a range of neovascular conditions in the eye, more targeted, cell-specific therapies may be more efficacious while minimizing adverse effects on physiologic angiogenesis.

### Current Treatments for Ocular Angiogenesis

Current anti-VEGF pharmacotherapies include humanized full-length monoclonal antibodies (bevacizumab), antibody fragments (ranibizumab, brolucizumab), and recombinant decoy receptors (aflibercept) (Table 1) (Todorich et al., 2014). Intravitreal injections are usually given in outpatients settings, but with frequencies as often as every 4–12 weeks, these treatments are costly and burdensome for patients (Suzuki et al., 2014; Cabral et al., 2017). Early success with off-label use of intravitreal bevacizumab led to the development of ranibizumab, which was designed to better penetrate the neurosensory retina and reduce systemic exposure based on its smaller size (48 kDa) (Heier et al., 2006), and received FDA approval for nAMD in 2006 following its success in phase 3 clinical trials (Rosenfeld et al., 2006; Kaiser et al., 2007; Brown et al., 2009). Since then, it has expanded its applications to diabetic macular edema, retinal vein occlusion-related macular edema, myopic CNV, and diabetic retinopathy, although it has not demonstrated superiority over the lower cost bevacizumab in randomized, prospective studies (CATT Research Group et al., 2011). Later studies led to the approval of aflibercept, which has demonstrated non-inferiority to ranibizumab for most of these indications, with the exception of DME in which aflibercept showed better short-term visual outcomes in eyes with poor baseline vision (Heier et al., 2014; Korobelnik et al., 2014a,b; Clark et al., 2016). The most recently-approved brolucizumab is the smallest (28 kDa) in size, and may exhibit greater durability due to the higher achievable therapeutic molar dose, but may also trigger more intraocular inflammation that could limits its wide adoption (Baumal et al., 2020; Dugel et al., 2020). Other anti-angiogenic pharmacotherapies under investigation include pegylated anti-VEGF designed ankyrin repeat proteins (abicipar pegol), antibodies against Tie-2 receptor ligands (faricimab, nesvacumab, ARP-1536), and PDGF antagonists (ranucumab, X-82) (Shen et al., 2014; Frye et al., 2015; Callanan et al., 2018; Sahni et al., 2019; Cohen et al., 2020; Heier et al., 2020; Khanani et al., 2020).

**Table 1:** A summray of current anti-VEGF drugs.

**Generic name**	**Bevacizumab**	**Ranibizumab**	**Aflibercept**	**Brolucizumab**
Trade name	Avastin	Lucentis	Eylea	Beovu
Structure	Full length humanized monoclonal antibody	Fragmented humanized monoclonal antiboody	Fusion protein containing domains from VEGFR-1 and VEGFR-2	Humanized single-chain antibody fragment
Molecular mass	149 kDa	48 kDa	115 kDa	26 kDa
Mechanism of action	Binds all isoforms of VEGF- A	Binds all isoforms VEGF-A	Binds all isoforms of VEGF-A, VEGF-B, and PIGF	Binds all isoforms of VEGF-A
Clinical development status	Off-label use; not FDA approved for ophthalmic use	FDA approval for nAMD (2006), DME (2012), mCNV (2017), DR (2017)	FDA approval for nAMD (2011), DME (2014), DR (2019)	FDA approval for nAMD (2019)
Ocular half-life in humans	4.9 days (Moisseiev et al., 2014)	7.19 days (Krohne et al., 2012)	11 days (Do et al., 2020)	4.3 days (Caruso et al., 2020)

*VEGF, vascular endothelial growth factor; PIGF, placental growth factor; nAMD, neovascular age related macular degeneration, DME, diabetic macular edema, mCNV, myopic choroidal neovasculatization; DR, diabetic retinopathy*.

Another mode of therapy for ocular angiogenesis is photodynamic therapy (PDT), which combines intravenous delivery of a porphyrin-based photosensitizer (verteporfin) with focal low-intensity light exposure to trigger singlet oxygen release within the CNV, causing vascular occlusion and ablation of the lesion. In clinical trials, PDT with verteporfin reduces vision loss and CNV in eyes with nAMD [Treatment of Age-Related Macular Degeneration With Photodynamic Therapy (TAP) Study Group, 1999; Verteporfin in Photodynamic Therapy Study Group, 2001], but did not show benefit over ranibizumab monotherapy (Cartwright et al., 1990). Although largely supplanted by anti-VEGF therapy today, PDT remains an important treatment modality for chronic central serous chorioretinopathy (Fujita et al., 2015), choroidal hemangiomas (Tsipursky et al., 2011), and polypoidal choroidal vasculopathy (Koh et al., 2012).

## Gene Therapies for Ocular Angiogenesis

### Vectors for Ocular Gene Therapy

The pursuit of gene therapies for ocular angiogenesis was born out of a need for more sustained treatments to overcome the burden of repeated injections. Ocular gene therapy has gained renewed interest since the approval of the first retinal gene therapy using an AAV2 vector to express the RPE65 gene encoding a retinal isomerase for patients with type 2 Leber Congenital Amaurosis (Bainbridge et al., 2008, 2015; Maguire et al., 2008). Unlike adenoviruses, which has been largely abandoned due to its immunogenicity (Walther and Stein, 2000), AAVs are well-suited for human applications because they are non-pathogenic, replication-deficient, and exhibit low immunogenicity. Different serotypes of AAV combined with cell-specific promoters can target distinct retinal cell types. In murine and non-human primate retina, ganglion cells are mainly transduced with AAV2 and AAV8, while photoreceptors and RPE can be efficiently transduced with AAV2, AAV5, AAV7, AAV8, AAV9 (Auricchio et al., 2001; Hori et al., 2019). As the viral tropism can differ between species, however, pre-clinical animal studies may not directly translate directly to human trials. In human retina, Wiley et al. reported that AAV4 and 5 are most efficient at transducing photoreceptors, AAV4 for transducing ganglion cells and the inner nuclear layer, and AAV4 and AAV6 for RPE cells, although the authors acknowledged donor-to-donor and age-dependent difference in transduction efficiency (Wiley et al., 2018). AAVs have a limited packaging capacity (4.7 kb). Larger genes are more suitably transduced with lentiviral vectors (Yáñez-Muñoz et al., 2006), which has a larger carrying capacity (10 kb), but has a greater risk of insertional mutagenesis as it integrates into the host genome (Walther and Stein, 2000). Synthetic delivery platforms such as poly (lactic-co-glycolide) (PLGA) nanoparticles have good biocompatibility and low immunogenicity (Kapoor et al., 2015; Mir et al., 2017), but are typically less efficient at transducing retinal cells compared with viral vectors.

### Modes of Vector Delivery

Most current retinal gene therapies employ a subretinal injection to deliver the viral vector (Figure 1). This method involves a vitrectomy surgery during which a thin cannula is inserted through the retina to create subretinal bleb in which viral particles can interface directly with photoreceptors and RPE. This method enables efficient gene transfer (Stieger et al., 2009) and exhibits minimal immunogenicity due to retinal immune privilege (Peng et al., 2017). However, the surgical procedure is invasive and the therapeutic effect is limited to the focal area of the bleb (Stout and Francis, 2011). Intravitreal injections can be performed in outpatient clinic settings, and the injected agent can diffuse across the entire globe (Figure 1), but efficacy is limited by the internal limiting membrane (ILM) which serves as a barrier on the inner surface of the retina (Stout and Francis, 2011). Newer generations of AAV derived by directed evolution, such as the AAV2-7m8 serotype (Dalkara et al., 2013), may be required to enable efficient transgene expression after intravitreal delivery. More recently, our research team and others have demonstrated the effective delivery of AAV into the suprachoroidal space—a potential space between the choroid and the scleral wall of the eye (Figure 1) (Ding et al., 2019; Yiu et al., 2020a). Although suprachoroidal drug delivery using microneedles has shown some promise in human studies (Willoughby et al., 2018; Yeh et al., 2020), its utility for viral gene therapy remains unclear.

### Gene Therapy Strategies for Ocular Angiogenesis

Most current anti-angiogenesis gene therapy strategies employ a biofactory approach of transducing retinal cells with viral vectors to produce VEGF antagonists. Early studies using AAV expression of the soluble VEGF receptor sFlt-1 demonstrated long-term expression of sFlt-1 and reduction in CNV size without significant immune response in non-human primates (MacLachlan et al., 2011; Lai et al., 2012) and in phase 1 human studies (Heier et al., 2014; Rakoczy et al., 2015a), but showed no clear functional benefit in a phase IIa trial of 32 patients (Constable et al., 2016). Many of the enrolled patients in this study had previously been treated with multiple anti-VEGF treatments, so it was unclear if there was a ceiling effect where additional visual gains would have been limited. Also, among the 21 patients that received the treatment, 12 of them had pre-existing neutralizing antibodies against AAV2, although the authors found no clear correlation between these antibodies and therapeutic efficacy (Constable et al., 2016). It is worth noting that the impact of pre-existing neutralizing antibodies against the viral vector may depend on the delivery route. While the effectiveness of subretinal AAV2 did not appear correlated with pre-existing antibody titers in several studies (Bennett et al., 2012; Rakoczy et al., 2015b; Constable et al., 2016), intravitreal injections of AAV2 showed lower therapeutic efficacy when serum neutralizing antibodies were present (Heier et al., 2014), perhaps due to the greater immune privilege of the subretinal space. Nevertheless, intravitreal injections using the newer generation AAV2-7m8 vector (ADVM-022) has shown sustained expression of aflibercept for more than 12 months in non-human primates (Grishanin et al., 2019), and stabilized visual acuity and retinal anatomy in 10 of 12 human patients without rescue anti-VEGF treatments over 24 weeks in a phase I study (NCT03748784), although transient intraocular inflammation was noted (Boyer, 2020). More recently, interim analysis of the phase II study found that 9 of 12 patients did not require rescue injection for 54 weeks, and 6 of these 9 patients maintained their vision for 74 weeks without additional injections (http://investors.adverum.com/news-releases/news-release-details/adverum-biotechnologies-announces-positive-interim-data-cohorts). Another strategy employing subretinal AAV8 to express a monoclonal anti-VEGF antibody fragment (RGX-314) has been found to be comparable to anti-VEGF Fab expression (Liu et al., 2018), and interim assessment of the phase I/IIa trial in nAMD patients also showed sustained expression (NCT03066258).

## Crispr-Based Approaches for Ocular Angiogenesis

### Genome Editing Using CRISPR-Cas9 Endonucleases

Most current anti-angiogenic gene therapy strategies only mimic pharmacologic VEGF inhibition. Thus, they do not affect intracellular targets and do not distinguish pathologic from physiologic cellular sources. Rather than targeting angiogenic factors at the protein or RNA level, which require transgene expression for sustained activity, genome editing using CRISPR-based systems enables modifications at the DNA level, providing (1) permanent suppression of angiogenic signals, (2) potential disruption of both extracellular and intracellular targets, and (3) possible cell-specific delivery aimed at more pathologically relevant sources.

Derived from prokaryotic adaptive immune systems, CRISPR-associated Cas9 endonucleases can induce a site-specific cleavage in the target DNA with programmable guide RNAs (gRNAs), creating double-strand breaks (DSBs) that can be repaired by error-prone non-homologous end-joining (NHEJ) or homology directed repair (HDR) when paired with donor DNA template (Yiu, 2018; Rodríguez-Rodríguez et al., 2019). The fast-moving technology now includes a compendium of different Cas orthologs from various bacterial and archaeal species, including engineered CRISPR-Cas proteins that enable gene repression without modifying DNA. For example, fusion of deactivated Cas9 lacking its catalytic domain with Kruppel Associated Box transcription repressor domains (CRISPRi) enables RNA-guided gene repression (Gilbert et al., 2013; Mandegar et al., 2016; Cox et al., 2017; Kim et al., 2017a). Cas13 is another CRISPR endonuclease that differs from Cas9 in that it targets RNA instead of DNA, and can knockdown mRNA transcripts with similar efficacy and fewer off-target effects than RNA interference (Abudayyeh et al., 2017, 2019). Fusion of catalytically deactivated Cas13 (dCas13) with Adenosine Deaminase Acting on RNA (ADAR) enables more precise RNA base editing (Cox et al., 2017; Abudayyeh et al., 2019). While these systems showed effective repression of target genes in mammalian cells without modulating DNA (Cox et al., 2017; Thakore et al., 2018; Abudayyeh et al., 2019; Chung et al., 2019; Truong et al., 2019), they have not yet been extensively applied to ocular angiogenesis.

### CRISPR Delivery to Ocular Tissues

While the Cas9 from *Streptococcus pyogenes* (SpCas9) has been the most well-characterized ortholog, its larger size (4.2 kb) limits its ability to be packaged with gRNAs into a single AAV vector for *in vivo* use. Strategies to circumvent this limitation include packaging in lentiviral vectors, employing dual AAV vectors to express full-length SpCas9 separately from the gRNAs, or using “split-Cas9” by dividing the expression of SpCas9 at its disordered linker (V713–D718) and reconstituting the full-length protein by split-intein protein trans-splicing (Chew et al., 2016). In addition, smaller Cas9 orthologs from *Staphylococcus aureus* (SaCas9) and *Campylobacter jejuni* (CjCas9) can be packaged along with gRNAs in an “all-in-one” AAV vector (Kim et al., 2017; Chung et al., 2020). The first human clinical trial utilizing CRISPR technology in the eye commenced in late 2019 and evaluates subretinal AAV-mediated delivery of SaCas9 with a pair of gRNAs to target a deep intronic mutation in the CEP290 gene for the treatment of type 10 Leber congenital amaurosis (NCT03872479) (Maeder et al., 2019). Interestingly, despite the theoretical advantage of using a single viral vector for clinical translation, various groups including ours have found that genome editing efficiency of these smaller Cas9 variants are inferior to dual-vector delivery of SpCas9 and gRNAs (Chung et al., 2020; Li, F. et al., 2020).

Although viral vectors allow efficient transfer of genome editing tools to retinal cells, sustained viral expression of the Cas9 endonuclease can lead to off-target effects. Unlike conventional gene augmentation or biofactory strategies, CRISPR systems do not require long-term transgene expression, where the sustained presence of Cas9 can potentially trigger non-specific mutations. An alternative strategy for clinical application is to directly deliver recombinant Cas9 proteins and gRNAs as ribonucleoprotein complexes (RNPs) to the eye, which can induced DNA cleavage almost immediately and degrade rapidly in cells, helping to minimize off-target effects and cellular toxicity (Kim et al., 2014; Jo et al., 2015). Direct application of RNPs to human cells demonstrated more efficient gene cleavage than plasmid transfection, with up to 79% on-target mutation and minimum off-target effects (Kim et al., 2014), and when delivered subretinally into mouse eyes to target VEGF, results in up to 40% reduction in a laser-induced model of CNV (Kim et al., 2017b). Despite the benefits of RNP, nuclear delivery of Cas9 proteins is still challenging, mainly due to endosomal entrapment in cytosol. Synthetic vehicles such as cell penetrating peptides (CPPs) may increase delivery efficiency by up to 80% (Zuris et al., 2015).

### Genomic Targets for Ocular Angiogenesis

As mentioned before, an advantage of using CRISPR technology over current anti-angiogenesis gene therapy approaches is the ability to target both extracellular cytokines and intracellular mediators including trans- and cis-regulatory elements. Beside targeting VEGF, for example, genome editing strategies could be designed to target the hypoxia-inducible factor 1α (Hif-1α) transcription factor, or the hypoxia response element (HRE) in the VEGF promoter to which Hif-1α binds. CRISPR-Cas9 may also target VEGF receptors or downstream signals. Finally, CRISPR-based strategies enable simultaneous and multiplexed targeting of several factors using an array of gRNAs (Cong et al., 2013; Mali et al., 2013b; Zhang et al., 2019), which can potentially make genome editing particularly well-suited for multifactorial conditions such as ocular angiogenesis. Thus, the design of CRISPR-based anti-angiogenesis therapies can be more sophisticated, more effective, and more specific than pharmacologic and conventional gene therapies. Here, we summarize efforts using genome editing to target various pro-angiogenic signals in preclinical models of ocular angiogenesis (Table 2).

**Table 2:** A summary of CRISPR technology suppressing pathologic ocular proangiogenic factors.

**Target gene**	**Experiments**	**Nuclease used**	**Delivery method**		**Results**	**References**
			**Vectors**	**Route**		
VEGF-A	Human RPE (ARPE-19) cell line	SpCas9	Lentivirus (single vector)	*in vitro*	37.0% indels and 41.2% VEGF suppression	Yiu et al., 2016
	Mouse model with laser-induced CNV	SpCas9 and SaCas9	AAV8 (dual vectors for SpCas9 and single vector for SaCas9)	Subretinal	30 and 17% reduction in CNV size SpCas9 and SaCas9, respectively	Chung et al., 2020
	Mouse retina; FACS sorted RPE cells	SpCas9	Lentivirus (single vector)	Subretinal	84% indel formation	Holmgaard et al., 2017
	Mouse model with laser-induced CNV	SpCas9	Cas9 RNP	Subretinal	22% indels and 40% reduction in CNV size	Kim et al., 2017b
	Mouse model with laser-induced CNV	CjCas9	AAV9 (single vector)	Intravitreal	20 and 22% indels in retina and RPE cells, respectively, and 20% reduction in CNV size	Kim et al., 2017
	Mouse model with laser-induced CNV	LbCpf1	AAV9 (single vector)	Intravitreal	57.2% indels in retina and 42% reduction in CNV size	Koo et al., 2018
VEGFR2 (KDR)	HRECs with cell specific promoter ICAM2	SpCas9	AAV5 (dual vectors)	*in vitro*	80% indels and 80% VEGFR2 suppression	Wu et al., 2017
	HRECs	SpCas9	Lentivirus (single vector)	*in vitro*	83.57% indels and HREC suppression	Huang et al., 2017b
	Vascular endothelial cells (ECs) with cell specific promoter ICAM2	SpCas9	AAV1 (dual vectors)	*in vitro*	80% reduction of VEGFR2 in EC	Huang et al., 2017a
	Mouse model of oxygen-induced retinopathy and laser-induced CNV	SpCas9	AAV1 (dual vector)	Intravitreal	30% suppression of VEGFR2 in mouse retina	
Hif1-α	Mouse model with laser-induced CNV	LbCpf1	AAV9 (single vector)	Intravitreal	59.2% indels in retina and 34% reduction in CNV size	Koo et al., 2018
	Mouse retina and RPE cells	CjCas9	AAV9 (single vector)	Intravitreal	58 and 31% indels, in retina and RPE cells, respectively, with 24% reduction in CNV size	Kim et al., 2017; Jo et al., 2019

*CRISPR, clustered regularly interspaced short palindromic repeats; VEGF, vascular endothelial growth factor; VEGFR, VEGF receptor; Hif1-α, hypoxia-inducible factor 1-α; RPE, retinal pigmented epithelium; HRECs, human retinal microvascular endothelial cells; ICAM2, intercellular adhesion molecule 2; CNV, choroidal neovascularization; Cas, CRISPR-associated; AAV, adeno-associated virus; RNP, ribonucleoproteins*.

#### VEGF

Our group successfully employed SpCas9 to target exon 1 of VEGF-A in human RPE cells using lentiviral vectors *in vitro*, with up to 37% indel formation, 41% reduction in VEGF-A protein, and 39% reduction in endothelial cell tube formation (Yiu et al., 2016). Holmgaard et al. later tested lentiviral-mediated SpCas9 delivery in the mouse retina, targeting exon 3 instead of exon 1, and achieved up to 84% VEGFa knockdown in mouse RPE cells (Holmgaard et al., 2017). Due to safety concerns related to the use of lentiviral vectors, Kim and colleagues subretinally injected preassembled Cas9 RNPs directly, and reduced VEGFa in RPE and laser-induced CNV by ~40% (Kim et al., 2017b). The same group also performed intravitreal AAV9 injections to deliver the smaller CjCas9 ortholog (Kim et al., 2017) and the type V endonuclease Cpf1 (also known as Cas12a) from *Lachnospiracea bacterium* (LbCpf1) which creates staggered DNA cuts (Koo et al., 2018), reducing laser-induced CNV area in mouse eyes by 24 and 42%, respectively. The mechanism by which intravitreal AAV in these studies effectively penetrated the retina to suppress CNV is unclear, although laser injury may have disrupted the ILM barrier. Interestingly, although some reports suggest that co-delivery of two AAV vectors may be less efficient than a single AAV (Trapani et al., 2014), our group found that dual AAV8 delivery of SpCas9 and gRNAs resulted in higher on-target editing rates *in vivo*, greater VEGF protein reduction, and more effective CNV suppression in mouse eyes than SaCas9 expression using a single AAV vector (Chung et al., 2020). In addition to DNA editing, Zhou et al. (2020) also utilized CasRx (RfxCas13d) to target VEGFa mRNA in the mouse retina demonstrating efficient VEGF knock down and CNV suppression using a paired gRNA system. Due to differences in CRISPR nucleases, gRNA design, mode of ocular delivery, and methods for quantifying efficacy, comparisons between studies are difficult. Nevertheless, these results show that VEGF protein reduction does not scale linearly with functional CNV suppression, and that despite varying levels of genomic VEGF disruption, CNV suppression in rodent models rarely exceeds 50%. This is supported by the fact that widely-used and clinically-effective pharmacologic anti-VEGF agents such as aflibercept achieve similar efficacies in laser CNV animal models (Koo et al., 2018; Chung et al., 2020). Thus, despite concerns that genome editing may not achieve the same high levels of VEGF inhibition as pharmacologic agents, they may still be effective in clinically settings.

#### VEGF Receptors

VEGF-A regulates angiogenesis through the tyrosine kinase receptors VEGFR-1 (Flt-1) and VEGFR-2 (KDR). Although VEGFR-1 has higher binding affinity, VEGFR-2 has greater kinase activity and mediates most of the pro-angiogenic signal (Shibuya, 2011). Early gene therapies employed AAV to express soluble VEGFR-1 (sFlt-1) as a decoy receptor, but showed limited efficacy in human trials (MacLachlan et al., 2011; Lai et al., 2012; Rakoczy et al., 2015; Constable et al., 2016). For genome editing strategies, VEGFR-2 has been the target of choice. But unlike VEGF which is secreted by multiple cell types, VEGF receptor knockdown must be targeted to endothelial cells. A dual AAV5 system expressing SpCas9 under the endothelial cell-specific promoter ICAM2 successfully depleted VEGFR-2 by 80% and reduced *in vitro* angiogenesis from human retinal microvascular endothelial cells (HREC) (Wu et al., 2017). A lentiviral vector carrying SpCas9 to target VEGFR2 also showed over 80% indel formation and HREC suppression *in vitro* (Huang et al., 2017b). The same group later demonstrated intravitreal AAV1-mediated suppression of VEGFR2 in both an oxygen-induced retinopathy (OIR) model of retinal neovascularization and laser-induced CNV (Huang et al., 2017a). Although these data support the potential benefit of targeting VEGF receptors, the pathway for clinical translation remains unclear. AAVs have natural tropism for neurons, skeletal muscle, and hepatocytes but not for vascular endothelium, although viral capsid modifications may enable greater transduction efficiency in these cells (Nicklin et al., 2001; Körbelin et al., 2016). Subretinal AAV also lack access to choroidal endothelial cells due to the RPE and Bruch's membrane, although disruption of this barrier by CNV pathology may overcome this limitation.

#### Hif-1α

Hif-1α is a major regulator of the cellular hypoxic response (Zimna and Kurpisz, 2015), and transcriptionally activates a variety of pro-angiogenic factors, chemokines, and receptors including VEGF, PDGF-B, and angiopoietins 1/2 (Semenza, 2003; Ceradini et al., 2004; Greijer et al., 2005; Manalo et al., 2005). AAV9 delivery of CjCas9 with gRNAs to target Hif-1α demonstrated ~20% CNV suppression in mouse eyes (Kim et al., 2017), with no detectable toxicity or off-target effects up to 14 months after treatment (Jo et al., 2019). Interestingly, the authors noted cone dysfunction in eyes that underwent genomic disruption of VEGF but not Hif-1α, implicating the latter as a safer therapeutic target. The same team also utilized LbCpf1 to knockdown Hif-1α in mice, with up to 34% reduction of laser-induced CNV (Koo et al., 2018). While the ability to target upstream regulatory factors such as Hif-1α appears attractive, there are also potential risks. Hif-1α mediates various pathways involved in physiologic, as well as pathologic, angiogenesis. Thus, permanent suppression of high-level regulators such as Hif-1α may have unintended adverse consequences, and may explain the limited use of pharmacologic Hif-1α antagonists such as doxorubicin in clinical ophthalmic practice.

## Limitations of Crispr for Ocular Angiogenesis

### Therapeutic Thresholds of Pro-Angiogenic Targets

Genome editing strategies face a unique set of challenges when applied to the treatment of angiogenic conditions. In inherited retinal diseases where gradual photoreceptor loss leads to eventual blindness, only 10% of photoreceptors need to be preserved or rescued to restore useful vision (Geller and Sieving, 1993; Ratnam et al., 2013). However, the therapeutic threshold for VEGF or other pro-angiogenic factors are unknown, and likely varies significantly between disease processes and individual patients. In humans, VEGF levels in the aqueous humor poorly predict disease severity in eyes with diabetic macular edema (Kwon and Jee, 2018). Variable responses to anti-VEGF pharmacotherapies are evidenced by many patients who do not respond to treatment. Because the laser-induced CNV model is notoriously unreliable as a predictor of clinical efficacy in humans, the relative effectiveness of genome editing strategies cannot be reliably compared with anti-VEGF pharmacotherapies in these animal models.

Additionally, the stochastic nature of CRISPR-based approaches limits our ability to carefully titrate the degree of angiogenic suppression. For example, Cas9 cleavage generates a mosaic of biallelic null mutants, haploinsufficient, and unedited wild-type cells, thus causing variable and incomplete VEGF suppression. The use of multiple gRNAs can increase the amount of gene knockdown (Tsai et al., 2018), but over-suppression may also be detrimental as VEGF is required for the normal, physiologic maintenance of vascular and neural tissues (Kurihara et al., 2012), and chronic anti-VEGF treatments have been linked to geographic or choroidal atrophy in patients with AMD and DME (Yiu et al., 2014; Grunwald et al., 2015). Thus, partial suppression of angiogenic pathways over the long-term may actually be preferred for clinical applications. For example, lentiviral delivery of VEGF-A-shRNA which reduces VEGF-A to physiologic levels rescues OIR in rats without interfering with retinal vascular development (Wang et al., 2013). Also, while current anti-VEGF therapies involve repeated, pulsatile treatments that result in fluctuations in VEGF suppression; continuous, stable VEGF suppression using sustained delivery systems appear effective even at lower therapeutic doses (Campochiaro et al., 2019).

Finally, it is important to note that pathologic angiogenesis involves the complex interaction of many pro- and anti-angiogenic factors (Cabral et al., 2017). Although pharmacologic VEGF inhibition appears to be effective across a spectrum of neovascular retinal diseases, genome editing produces a cellular mosaic of homozygous null mutants which could be impacted by compensatory paracrine effects from neighboring cells or upregulation of other proangiogenic pathways.

### Cell Specificity of CRISPR-Based Therapies

Unlike gene therapies that employ a biofactory approach, CRISPR-mediated strategies must be appropriately directed at pathologic cell types. For example, genomic VEGF ablation in Muller glia may be more suitable for treating retinal neovascularization, while VEGF suppression from RPE or choroidal endothelial cells may be more appropriate for CNV. Similarly, genomic disruption of the VEGF receptor must be targeted to retinal or choroidal endothelial cells depending on the nature of the disease, although recent evidence suggests that retinal vessels may also contribute to CNV pathogenesis (Snyder et al., 2018; Yiu et al., 2019; Lee et al., 2020). Target cell specificity may be conferred by cell-specific promoters, AAV serotypes with different cellular tropisms, or distinct modes of vector delivery. For example, subretinal injections are more likely to effectively treat a CNV lesion, while intravitreal injections may be better suited for widespread retinal neovascular diseases. Although cellular targeting may be particularly challenging for certain cell types in the retina, these considerations could enable greater precision and safety profile of CRISPR-based treatment platforms.

### Minimization of Off-Target Activity

Because genome editing therapies are optimally prescribed prior to the onset of severe vision loss, the prospect of potentially inducing unintended mutations is of particular concern. Strategies to minimize off-target genome editing activity include truncated gRNAs, high fidelity Cas9 variants, anti-CRISPR proteins, and self-destructing CRISPR systems. Several studies have shown that truncated gRNAs (<20 nt in length) reduce off-target activity without compromising on-target specificity by minimizing their binding affinity to DNA (Pattanayak et al., 2013; Fu et al., 2014; Tsai et al., 2015). Utilizing Cas9 nickases with paired gRNAs also limit off-target effects, as individual off-target sites of single-stranded nicks are rapidly repaired by base excision mechanisms (Mali et al., 2013a; Ran et al., 2013; Cho et al., 2014). Newly-engineered high fidelity Cas9 variants such as SpCas9-HF1 (Kleinstiver et al., 2016) and high fidelity SaCas9 (Tan et al., 2019; Xie et al., 2020) also showed promise in reducing non-specific mutations. By binding to SpCas9's protospacer adjacent motif (PAM) recognition sites, small anti-CRISPR proteins (Acr) less than 200 amino acids can be delivered via AAV vectors to suppress genome editing activity (Shin et al., 2017). *In vivo* AAV8 delivery of AcrIIC3 with Nme2Cas9 in mice inhibited genome editing without demonstrating cytotoxicity (Lee et al., 2019), although the optimal timing of Acr delivery remain to be determined. Finally, self-destructing “kamikaze” CRISPR systems employ gRNAs that target the Cas9 endonuclease itself to limit prolonged genome editing activity (Li et al., 2018). Although dual AAV2-mediated expression of SpCas9 and gRNA targeting SpCas9 successfully reduced SpCas9 mRNA after intravitreal delivery (Li et al., 2019), concerns of incomplete Cas9 removal may limit the usefulness of these approaches (Li et al., 2018). Given the unique optical system of the eye as an organ, optogenetically-controlled nanoCRISPR technology could enable spatial and temporal control of Cas9 endonuclease activity to improve safety and precision in future studies (Chen et al., 2020).

### Immune Responses to CRISPR Components

The foreign nature of bacterial-derived CRISPR-Cas9 proteins has the potential to elicit unwanted host immune responses that may limit therapeutic efficacy. In mice, AAV-mediated Cas9 delivery triggered both humoral and cellular response against Cas9 (Chew et al., 2016). Moreover, mice with preexisting antibodies against SaCas9 evoked CD8^+^ cytotoxic T cell activity which eliminated the edited cell population despite still showing efficient genome editing (Li et al., 2020). In humans, pre-existing anti-Cas9 antibodies and antigen-reactive T cells are prevalent due to the ubiquitous nature of *S. pyogenes* and *S. aureus* from which the most commonly-used SpCas9 and SaCas9 endonucleases are derived (Attenello, 2019; Charlesworth et al., 2019; Wagner et al., 2019). Studies in human blood donors revealed pre-existing antibodies against SaCas9 and SpCas9 ranging from 78–79 to 58–65%, respectively (Attenello, 2019) (Charlesworth et al., 2019). Interestingly, effector T cell responses have been found in donor peripheral blood monocytes (PBMCs) upon restimulating with recombinant Cas9s, suggesting that adaptive immunity may lead to diminishing therapeutic efficacy (Wagner et al., 2019). Although antibodies against intracellular proteins may not directly lead to immune-mediated elimination of Cas9-expressing cells (Crudele and Chamberlain, 2018), these pre-existing antibodies can limit the efficacy of the CRISPR gene therapy.

## Conclusion

The application of genome editing for retinal diseases has received significant attention since the recent initiation of the first human trial using CRISPR technology. Genome editing pose unique advantages as well as challenges when compared to anti-VEGF pharmacotherapies or current gene therapy approaches. Genomic ablation can permanently suppress pro-angiogenic pathways, providing a true cure for ocular angiogenic conditions. Viral-mediated delivery of CRISPR components also enables efficient targeting of not only secreted factors such as VEGF, but also intracellular targets including upstream transcription factors and regulatory elements, as well as downstream signal mediators. Yet, the needs to overcome a high therapeutic threshold and maximize cellular specificity, while minimizing off-target activity and host immune responses, must be adequately addressed to facilitate more rapid translation of research outcomes into real world therapies.

## Author Contributions

SC and GY conceived the project. SC, T-NS, and TN collected literature. SC, T-NS, and GY wrote the manuscript. GY oversaw the project. All authors contributed to the article and approved the submitted version.

## Conflict of Interest

The authors declare that the research was conducted in the absence of any commercial or financial relationships that could be construed as a potential conflict of interest.
